# Utility of Non-Invasive Monitoring of Cardiac Output and Cerebral Oximetry during Pain Management of Children with Sickle Cell Disease in the Pediatric Emergency Department

**DOI:** 10.3390/children5020017

**Published:** 2018-02-01

**Authors:** Pradeep Padmanabhan, Chikelue Oragwu, Bibhuti Das, John A. Myers, Ashok Raj

**Affiliations:** 1 Division of Pediatric Emergency Medicine, Mease Countryside Hospital, Safety Harbor, FL 34695, USA; 2 Medical Affairs, Virology, Merck & Co., Inc., Marietta, GA 30067, USA; drchikelue@gmail.com; 3 Joe DiMaggio Children’s Hospital, Memorial Health Care, Hollywood, FL 33021, USA; bdas@mhs.net; 4 Department of Pediatrics, University of Louisville, Louisville, KY 40202, USA; John.myers@louisville.edu; 5 Division of Pediatric Hematology/Oncology, Department of Pediatrics, University of Louisville, Louisville, KY 40202, USA; ashok.raj@louisville.edu

**Keywords:** sickle cell disease, pain crisis, electrical cardiometry, near-infrared spectroscopy

## Abstract

Pain crisis in children with sickle cell disease (SCD) is typically managed with intravenous fluids and parenteral opioids in the pediatric emergency department. Electrical cardiometry (EC) can be utilized to measure cardiac output (CO) and cardiac index (CI) non-invasively. Near-infrared spectroscopy (NIRS) measuring cerebral (rCO_2_) and splanchnic regional (rSO_2_) mixed venous oxygenation non-invasively has been utilized for monitoring children with SCD. We studied the value and correlation of NIRS and EC in monitoring hemodynamic status in children with SCD during pain crisis. We monitored EC and NIRS continuously for 2 h after presentation and during management. Forty-five children participated in the study. CO (D = 1.72), CI (D = 1.31), rSO_2_ (D = 11.6), and rCO_2_ (D = 9.3), all increased over time. CO max and CI max were achieved 1 h after starting resuscitation. rCO_2_ max attainment was quicker than rSO_2_, as monitored by NIRS. CI max correlated with rCO_2_ max (*r* = −0.350) and rSO_2_ max (*r* = −0.359). In adjustment models, initial CI significantly impacted initial rCO_2_ (*p* = 0.045) and rCO_2_ max (*p* = 0.043), while initial CO impacted rCO_2_ max (*p* = 0.030). Cardiac output monitoring and NIRS monitoring for cerebral and splanchnic oxygenation were feasible and improved the monitoring of therapeutic interventions for children with SCD during pain crisis.

## 1. Introduction

Pain crises account for 79–91% of pediatric emergency department (PED) visits and up to 70% of hospitalizations in patients with sickle cell disease (SCD) [1]. In 2004, the Agency for Healthcare Research and Quality reported that nearly 80% of the 80,000 hospital admissions for SCD began their course in the emergency department (ED) [2,3]. Vaso-occlusive crisis from obstruction to blood flow, impaired oxygen delivery, tissue hypoxia, ischemia, and infarction, may result in significant morbidity and mortality [4]. Treatment for pain crisis in the ED typically consists of intravenous (IV) fluid hydration and analgesia to provide prompt symptomatic relief. However, children with SCD have been found to have left ventricular (LV) dilatation and dysfunction when compared to age matched controls [5]. Therefore, rapid and excessive IV hydration without monitoring cardiac hemodynamics could result in heart failure and pulmonary edema. There are caveats to conventional measurements, such as pulse-oximetry (dependent on hemoglobin levels which are decreased and vary in SCD), and the typical tachycardia in pain crisis could be compounded by factors such as anemia and pain, and may not be attributable to dehydration alone. While the complications of the management of children with pain crisis from SCD are low in a carefully controlled, monitored setting, additional focused monitoring may provide useful information.

Non-invasive measurements of hemodynamic status, particularly cardiac output (CO), have been studied and found to be useful in SCD children undergoing plasmapheresis [6]. Children with SCD with significant hemodynamic changes during erythrocytapheresis, including hypotension, have been studied using non-invasive cardiac output monitoring [5]. Electrical cardiometry (EC) has been found to correlate well with the directly measured CO derived by the Fick oxygen principle in children with various congenital heart diseases [7]. Cerebral oximeter is a non-invasive diagnostic modality based on the principle of near-infrared spectroscopy (NIRS), which gives an indication of cerebral oxygenation. It has been proven to measure cerebral oxygenation to recognize disease severity, along with improvements noted in SCD children receiving transfusion therapy [8].

We hypothesize that the monitoring of hemodynamics and tissue oxygenation for SCD patients while receiving IV fluid and analgesics for pain crisis, will be feasible and add valuable information. Therefore, we sought to evaluate the utility of non-invasive monitoring of CO by EC and monitoring of cerebral oxygenation by cerebral oximeter and splanchnic oxygenation by NIRS in children with SCD presenting with pain crisis in a PED setting.

## 2. Materials and Methods

This was a prospective, observational, convenience sample study of pediatric patients with SCD presenting to the ED. After approval from the institutional review board of the University of Louisville, School of Medicine, children with SCD aged 3–18 years who were treated for pain crisis at Norton Children’s Hospital ED, Louisville, KY, from May 2010 to December 2013 were included in the study. These patients were considered high priority at the ED, ensuring the facilitation of rapid assessment and management. All subjects received the standard management of sickle cell pain crisis with administration of IV fluids (20 mL/kg of normal saline, max 1000 mL over one hour) and analgesia—morphine 0.1–0.15 mg/kg with repeat doses 50–100% of original dose depending on pain control.

The exclusion criteria included subjects with pre-existing neurological conditions such as cerebral palsy or developmental delay, cyanotic heart disease, or history or risk of an adverse skin reaction due to dermatological hypersensitivity. Patients who were suspected to have hypoxemia (less than 90% on pulse oximetry) due to concurrent infections or comorbidities other than sickle cell pain crisis were also excluded.

Once subjects met the eligibility criteria, informed and written (when applicable) consent was obtained. Relevant clinical data including age, weight, height, pain duration, pain score, home medications, baseline mental status, vital signs, perfusion status (capillary refill, skin turgor), fever, chest pain, presence/absence of difficulty breathing, and initial pulse oximetry were obtained. All components of ED evaluation and treatment, including laboratory data, medications, and IV fluids, were recorded using a standardized data collection form. Cardiac output (using EC from ICON™ CO monitor, Cardiotronic-Osypka Medical, La Jolla, CA, USA), cerebral oximetry (using INVOS cerebral oximeter 4100, Covidien-Medtronic, Minneapolis, MN, USA), and splanchnic oxygenation measured using NIRS probe were measured continuously for two hours. These measurements were recorded every 15 min for 2 h after initiation of IV fluids and/or analgesics. Age-appropriate pain scores on a scale of 1–10 (Numerical Pain Scale) [9,10] were also obtained every 15 min for the 2 h duration and the Wong–Baker Faces pain rating scale was used for children who could not verbalize pain.

### Statistical Analysis

The current study aimed to examine the impact of IV fluids and parenteral opioids on CO, CI and oxygenation (rCO_2_ and rSO_2_), over time, for pediatric SCD patients experiencing a pain crisis. To realize this goal, *n* = 45 pediatric SCD patients who experienced a pain crisis and were administered intravenous IV fluids and parenteral opioids, were monitored during their management. Initially, a descriptive analysis was performed on the demographic data, as well as on the baseline physiological disease markers and associated outcomes. Subsequently, repeated-measures ANOVA techniques were used to test if intravenous IV fluids and parenteral opioids affected outcomes over time. Poisson regression techniques were used to measure the rate of change of each measuring parameters rates over time (e.g., increase in CO per minute). The test significance level was set at 0.05 and no alpha-splicing was performed since each output CO was viewed as important in isolation. Lastly, exploratory analyses were conducted, in which generalized estimating equation (GEE) models where pain scores were made a function of time and disposition status, were developed.

## 3. Results

The majority of the 45 subjects were male (*n* = 25, 55.6%) with a mean age of 12.1 years (ages 10.8–13.4 years, SD = 4.4). Table 1 displays the measures studied at baseline and the end of follow-up. Cardiac Output (Δovertime = 1.72, *p* < 0.01), Cardiac Index (Δovertime = 1.31, *p* < 0.01), Splanchnic Oxygenation (Δovertime = 11.6, *p* < 0.01) and Cerebral Oxygenation (Δovertime = 9.3, *p* < 0.01), all increased over time.

The time to reach CO and maximum CI (CI max) max was approximately one hour (63.4 min vs. 63.7 min, *p* = 0.812). While it took over an hour to reach rSO_2_ max (70.0 min), it took less time to reach rCO_2_ max (44.3 min). The difference between the two times were significant (25.7 min, *p* = 0.003). Although the overall increase in rSO_2_ and rCO_2_ was similar (20.9% increase vs. 20.4% increase, *p* = 0.482), the rate at which these levels were reached was 59% higher for the cerebral area (1.62 per h vs. 1.02 per h, *p* < 0.001). That is, while both areas eventually reached similar levels, the improvement in cerebral oxygenation was significantly faster.

The correlations between the studied variables are presented in Table 2. Unadjusted, CI max was correlated with cerebral oxygenation max (*r* = −0.350, *p* = 0.020) and splanchnic oxygenation max (*r* = −0.359, *p* = 0.006). Unadjusted, time until splanchnic oxygenation max was correlated with initial CO (*r* = −0.304, *p* = 0.042), CO min (*r* = −0.317, *p* = 0.034), CO max (*r* = −0.294, *p* = 0.050), initial CI (*r* = −0.350, *p* = 0.018), CI min (*r* = −0.353, *p* = 0.017), and CI max (*r* = −0.351, *p* = 0.018).

In adjustment models (adjusting for age, gender, hemoglobin and hematocrit, initial CI significantly impacted initial rCO_2_ (*p* = 0.045) and rCO_2_ max (*p* = 0.043), while initial CO impacted rCO_2_ max (*p* = 0.030).

For the time to achieve CO max, the average was 72 min for the admitted group and 52 min for the discharged group (*p* = 0.13). For the time to achieve CI max, the average time was 70 min for the admitted group and 55 min for the discharged group (*p* = 0.22). 

**Figure 1:**
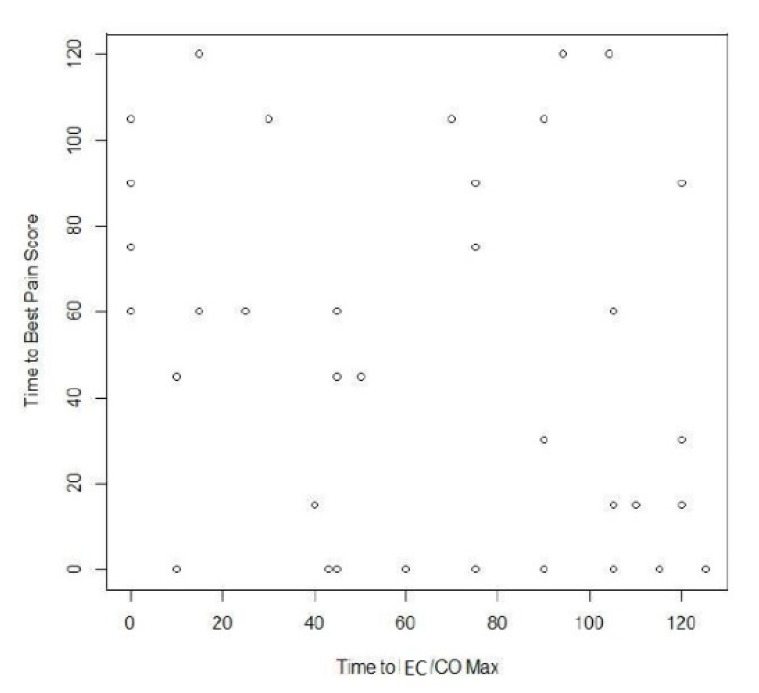
Time to best pain score vs time to Electrical Cardiometry/Cardiac Output max.

Pain scores improved along with CO/CI measurements. There was a significant difference (decrease) in pain scores over the first hour (*p* = 0.03). Pain scores trend lower with time (Figure 1) at least over the first hour.

## 4. Discussion

Our study, measuring cardiac output, cardiac index, cerebral and splanchnic oxygenation, utilizing non-invasive monitors, was feasible and well-tolerated by children with SCD presenting with pain crisis in a PED setting. During management of pain crisis in the PED, no significant negative impact was noted in cerebral/splanchnic oxygenation and CO during the time of monitoring. Also, the subjects did not have any clinical deterioration arousing suspicion, particularly heart failure or pulmonary edema.

The frequency of pain episodes in SCD and the need for hospital re-admission after ED visits have been associated with an increased risk of complications and death, suggesting that rapid and thorough evaluation and management of these episodes are needed [11,12,13]. Accurate assessment of pain using pain scales are important for effective pain management [14] as many times pain is underestimated in this vulnerable patient population. 

During management in the ED, all the values measured, in addition to standard parameters including cardiac outputCO, CI, rSO_2_ and rCO_2_ increased with time. The changes were maintained during the study period but we are unsure of their status beyond two hour. Maintenance of the usual hemodynamic parameters with mostly steady improvement in CO and CI measures, assure us of the safety of the parenteral opioid analgesics, which were administered to all study subjects.

Cerebral oxygenation, as measured by a cerebral oximeter, improved much earlier than splanchnic oxygenation, at a rate faster by almost 26 min. Interestingly, although one would assume that improvements in oxygenation would follow similar changes in CO/CI, maximal cerebral oxygenation (rCO_2)_) was approximately 18 min earlier than the attainment of peak CO. An established physiologic principle is that flow and oxygenation are selective towards vital organs and, in particular, the brain, yet the peak in rCO_2_ occurred much earlier, before the maximal CO. This could, in part, be because the cerebral oximetry monitoring based on the principles of NIRS measures mixed venous oxygen saturation, which reflects both oxygen delivery (like pulse oximetry) and oxygen utilization. In addition, the variation could be related to altered neuro-humoral reactions in SCD. Pain control combined with a decreased level of consciousness/induction of sleep (from opioid analgesics), will reduce oxygen utilization and improve tissue oxygen availability, which is detected by cerebral oximeter as an increase in cerebral oxygenation, similar to prior studies [15].

Cardiac output is dependent on four important elements: stroke volume, heart rate, preload, and afterload. As previously noted, children with SCD have baseline differences in cardiac hemodynamic parameters compared to healthy children. In SCD, echocardiography has demonstrated a higher CI at baseline [5,16]. Children with SCD have a higher preload, contributing to an increase in CI [5]. When children under general anesthesia were studied with additional CO monitoring, though very few adverse events were noted, a decrease in CI prior to a decrease in blood pressure or another parameter was noted during approximately 25% of such incidents [17]. This is in contrast to our study, where no such decreases in CO/CI were noted, perhaps due to the safety profile and careful dosing of IV opioid analgesics. In addition, all subjects received normal saline fluid administration. Due to the above-mentioned baseline differences compared to the normal population and the little understood cardiac hemodynamic changes under pain crisis in SCD children, trend monitoring strategies utilizing EC can and will have a vital role in monitoring children with SCD undergoing fluid resuscitation (e.g., in pain crisis) or during exchange blood transfusions.

The incidence of adverse events in pain crisis in SCD in well-controlled, tertiary care settings is low. While CI/CO changes may not have been significant in this study, other SCD-related serious complications, such as acute chest syndrome, and procedures, such as exchange transfusions, may be situations where such monitoring will be of significant value. The fact that the time to maximum CO/CI measurements by EC were shorter for discharged than for admitted patients, though not statistically significant, could be reflective of milder symptomatology in the discharged patients and the need for less aggressive hydration.

In conclusion, our study supports the current clinical practice of hydration and use of potent opiod analgesics in the management of sickle cell pain crises in the ED. Use of non-invasive monitoring with EC may be considered as an adjunctive in the assessment of patients, especially for those at risk of cardiovascular complications. Additional studies are required to see the impact of such monitoring on patient care, especially in the early identification of potentially critical issues.

## Figures and Tables

**Table 1:** Studied variables at baseline and at maximum level.

Variable	Baseline Mean (SD)	Maximum Level Mean (SD)	*p*-Value
Cardiac Output	5.15 (2.5)	6.87 (2.3)	<0.01
Cardiac Index	3.70 (1.3)	5.01 (1.2)	<0.01
Cerebral O_2_	49.8 (8.8)	59.1 (8.5)	<0.01
Splanchnic O_2_	60.4 (15.6)	72.0 (14.8)	<0.01
Hemoglobin	9.25 (1.9)	-	-
Hematocrit	26.63 (5.9)	-	-
Fluids Administered	0.85 (0.5)	-	-

**Table 2:** Correlation matrix of all variables studied.

Variable	Initial Cerebral Oxygenation	Minimum Cerebral Oxygenation	Maximum Cerebral Oxygenation	Time to Cerebral Oxygenation Max	Initial Splanchnic Oxygenation	Minimum Splanchnic Oxygenation	Maximum Splanchnic Oxygenation
Initial Cardiac Output	−0.125	−0.070	0.158	0.158	0.151	0.147	0.182
Minimum Cardiac Output	−0.058	−0.007	0.113	0.15	0.147	0.182	−0.064
Maximum Cardiac Output	−0.156	0.019	−0.067	−0.038	0.162	0.198	0.067
Time to Max Cardiac Output	−0.199	0.100	−0.016	−0.088	0.185	0.120	−0.021
Initial Cardiac Index	−0.193	−0.059	−0.048	−0.109	0.143	0.090	−0.006
Minimum Cardiac Index	0.088	0.037	0.100	−0.145	−0.014	−0.086	−0.123
Maximum Cardiac Index	−0.362	0.146	−0.350 *	0.006	0.141	0.111	−0.359
Time to Max Cardiac Index	−0.300	0.129	0.027	0.044	0.126	−0.128	−0.162
HB/HCT	−0.090	0.024	0.141	−0.005	−0.025	−0.150	0.019
HB	−0.107	−0.058	−0.068	0.148	−0.006	0.088	−0.048
HCT	−0.088	0.158	0.176	0.007	−0.193	−0.007	−0.109
Reticulocyte count	−0.166	0.170	0.161	−0.199	−0.070	−0.016	0.185
Fluids	−0.231	0.123	−0.156	0.165	−0.067	−0.088	0.198
Total White Blood Count	−0.083	0.073	0.123	0.213	−0.038	0.162	−0.115

* Significant at the 0.05 level.

## References

[1] Jacob E., Mueller B.U. (2008). Pain experience of children with sickle cell disease who had prolonged hospitalizations for acute painful episodes. Pain Med..

[2] Steiner C.A., Miller J.L., Agency for Health Care Policy and Research (US) Sickle Cell Disease Patients in US Hospitals, 2004: Statistical Brief #21. Healthcare Cost and Utilization Project (HCUP) Statistical Briefs (February 2006–December 2006). http://www.ncbi.nlm.nih.gov/books/NBK63489.

[3] Plarr O.S., Thorington B.D., Brambilla D.J., Milner P.F., Rosse W.F., Vichinsky E., Kinney T.R. (1991). Pain in sickle cell disease. Rates and risk factors. N. Engl. J. Med..

[4] Embury S.H. (1994). Sickle Cell Disease: Basic Principles and Clinical Practice.

[5] Poludasu S., Ramkissoon K., Salciccioli L., Kamran H., Lazar J.M. (2013). Left ventricular systolic function in sickle cell anemia: A meta-analysis. J. Card. Fail..

[6] Das B.B., Raj A., Recto M., Kong M., Bertolone S. (2012). Utility of impedance cardiography for the detection of hemodynamic changes in stable patients with sickle cell disease. J. Pediatr. Hematol. Oncol..

[7] Norozi K., Beck C., Osthaus W.A., Wille I., Wessel A., Bertram H. (2008). Electrical velocimetry for measuring cardiac output in children with congenital heart disease. Br. J. Anaesth..

[8] Raj A., Bertolone S.J., Mangold S., Edmonds H.L. (2004). Assessment of cerebral tissue oxygenation in patients with sickle cell disease: effect of transfusion therapy. J. Pediatr. Hematol. Oncol..

[9] Jensen M.P., Turner J.A., Romano J.M. (1994). What is the maximum number of levels needed in pain intensity measurement?. Pain.

[10] Pain Intensity Instruments National Institutes of Health—Warren Grant Magnuson Clinical Center, July 2013. http://www.mvltca.net/presentations/mvltca.pdf.

[11] Buchanan G., Vichinsky E., Krishnamurti L., Shenoy S. (2010). Severe sickle cell disease-Pathophysiology and therapy. Biol. Blood Marrow Transplant..

[12] Ballas S.K. (2005). Pain management of sickle cell disease. Hematol. Oncol. Clin. N. Am..

[13] Platt O.S., Brambilla D.J., Rosse W.F., Milner P.F., Castro O., Steinberg M.H., Klug P.P. (1994). Mortality in sickle cell disease. Life expectancy and risk factors for early death. N. Engl. J. Med..

[14] Todd K.H., Ducharme J., Choiniere M., Crandall C.S., Fosnocht D.E., Homel P., Tanabe P. (2007). PEMI Study Group. Pain in the emergency department: Results of the pain and emergency medicine initiative (PEMI) multicenter study. J. Pain.

[15] Padmanabhan P., Berkenbosch J.W., Lorenz D., Pierce M.C. (2009). Evaluation of cerebral oxygenation during procedural sedation in children using near infrared spectroscopy. Ann. Emerg. Med..

[16] Eddine A.C., Alvarez O., Lipshultz S.E., Kardon R., Arheart K., Swaminathan S. (2012). Ventricular structure and function in children with sickle cell disease using conventional and tissue echocardiography. Am. J. Cardiol..

[17] Coté C.J., Sui J., Anderson T.A., Bhattacharya S.T., Shank E.S., Tuason P.M., August D.A., Zibaitis A., Firth P.G., Fuzaylov G. (2015). Continuous noninvasive cardiac output in children: Is this the next generation of operating room monitors? Initial experience in 402 pediatric patients. Paediatr. Anaesth..

